# Large language models in emergency medicine education: opportunities, challenges, and implementation pathways

**DOI:** 10.3389/fpubh.2026.1899528

**Published:** 2026-08-05

**Authors:** Tingting Fan, Tianle Gao, Wan Tang, Qian Xu, Xingyou Wang, Qiaoli Su, Qingguo Lyu

**Affiliations:** 1 Emergency Center, Chengdu Integrated TCM & Western Medicine Hospital, Chengdu, China; 2 Department of Cardiovascular Medicine, Chengdu Integrated TCM & Western Medicine Hospital, Chengdu, China; 3 School of Health Policy and Management, Chinese Academy of Medical Sciences & Peking Union Medical College, Beijing, China; 4 General Practice Ward/International Medical Center Ward, General Practice Medical Center, West China Hospital, Sichuan University, Chengdu, China; 5 Wangjiang Hospital, Sichuan University, Chengdu, China; 6 Department of Endocrinology and Metabolism, West China Hospital, Sichuan University, Chengdu, China

**Keywords:** artificial intelligence, emergency medicine, large language model, medical education, residency training

## Abstract

**Background:**

Emergency medicine education occurs in high-acuity, interruption-prone, and time-constrained environments, where learners must develop rapid clinical reasoning, effective communication, procedural competence, and reliable documentation skills. Large language models (LLMs) are increasingly being explored in health-professions education. This narrative review synthesizes emerging applications, major risks, and implementation pathways for LLMs in emergency medicine education.

**Methods:**

A structured narrative review was conducted using PubMed, Web of Science Core Collection, China National Knowledge Infrastructure (CNKI), and Wanfang Data. The search period extended from January 1, 2023, to April 10, 2026. English and Chinese search blocks combined terms related to LLMs or generative artificial intelligence, emergency medicine or emergency care contexts, and education, training, simulation, assessment, communication, documentation, or implementation. After duplicate removal, title and abstract screening, and full-text review, 48 English-language studies and 5 Chinese-language studies were included. Eligible records addressed emergency medicine or emergency medical services education, simulation or virtual-patient applications, formative assessment and feedback, documentation or discharge communication, or governance issues relevant to educational use in emergency settings.

**Results:**

LLMs showed potential across multiple educational domains in emergency medicine, including just-in-time tutoring, resource generation, case drafting, simulation and virtual-patient rehearsal, formative feedback support, examination and competency-assessment support, documentation and discharge communication coaching, and educator workflow support. Potential benefits included more timely feedback, broader access to structured teaching resources, repeated rehearsal of low-frequency high-acuity scenarios, and greater consistency in communication training. Translation into routine educational practice remains constrained by hallucination, context mismatch with local protocols, automation bias, limited relational authenticity in AI-mediated interaction, privacy and cybersecurity concerns, multilingual inequity, uncertain validity of AI-assisted assessment, and uneven faculty readiness.

**Conclusion:**

LLMs hold substantial promise for strengthening emergency medicine education, particularly in areas requiring rapid language-based support, structured feedback, scalable case generation, and communication rehearsal. Current evidence supports phased adoption, local grounding in institutional protocols, secure workflows, explicit faculty oversight, and evaluation of educational, operational, and governance outcomes. This review proposes a pragmatic framework for the integration of LLMs into emergency medicine education.

## Introduction

Emergency medicine education occurs at the intersection of acute patient care, rapid decision-making, and unpredictable clinical complexity (1). Learners must synthesize incomplete information, prioritize under time pressure, perform procedures safely, communicate across professions, and document care while managing frequent interruptions and uncertainty (1, 2). Teaching in this setting is challenging because supervision time, feedback opportunities, and exposure to high-stakes cases are inherently uneven (1, 2). These constraints make emergency medicine education highly dependent on tools that can support rapid learning, structured communication, and timely feedback within busy clinical workflows (2).

Artificial intelligence has become increasingly prominent in scientific discovery, clinical medicine, undergraduate medical education, and healthcare education (3–5). This transition has been accelerated by foundation models and LLMs, which enable natural-language interaction with educational and clinical content through summarization, question answering, case generation, and dialogue simulation (6, 7). Generative artificial intelligence refers to systems that generate new content by learning statistical patterns from large datasets rather than by reasoning or understanding in human terms (4, 8, 9). In medical education, reported applications include multiple-choice question generation, interactive clinical simulations, rehearsal of difficult conversations, and curriculum design (10, 11). For time-constrained educators, these tools may reduce first-draft burden and improve access to structured teaching material, but they require expert review to preserve clinical accuracy and the humanistic core of teaching (10).

Previous reviews and empirical studies have described the rapid expansion of AI and LLM applications in emergency and critical care, including triage, acuity assessment, documentation, and operational support (12–15). In emergency medicine, the relevance of LLMs is particularly evident because many core teaching tasks are language mediated, including triage explanation, differential diagnosis discussion, handoff communication, discharge counseling, reflection, and debriefing (16). Emergency medicine is also a distinct educational context: training occurs in crowded and unpredictable clinical settings characterized by variable case mix, high-acuity decision making, and limited time for individualized coaching (1). Educational interventions therefore affect not only learning efficiency but also workflow reliability and patient-safety culture (16–18). These features make emergency medicine a particularly important setting for the educational use of LLMs in structured teaching support, feedback generation, communication training, and language-based cognitive scaffolding (16, 17).

Despite the rapid growth of the literature on LLMs in medical education and emergency care, focused synthesis of how these tools can be integrated into emergency medicine education, and under what safeguards, remains limited (10, 11, 16, 17). This narrative review therefore examines the emerging evidence on educational applications, major risk domains, and practical implementation pathways for LLMs in emergency medicine education.

## Methods

This structured narrative review was informed by SANRA principles for narrative reviews rather than designed as a PRISMA-style systematic review or meta-analysis (19). Four databases were searched: China National Knowledge Infrastructure (CNKI), Wanfang Data, PubMed, and Web of Science Core Collection. The search period extended from January 1, 2023, to April 10, 2026. Search blocks combined terms related to large language models or generative artificial intelligence, such as “large language model*,” “generative AI,” ChatGPT, and GPT; emergency-care contexts, such as emergency medicine, emergency department, and resuscitation; and educational targets, including education, training, and teaching.

In this review, “emergency medicine education” was operationally defined as LLM-supported teaching, learning, simulation, case rehearsal, feedback, assessment, communication training, documentation coaching, protocol learning, or preparedness training for emergency medicine learners, residents, faculty, EMS personnel, and other emergency-care professionals. Eligible records included original articles and reviews addressing one or more of the following topics: (1) emergency medicine or emergency medical services education; (2) resuscitation or emergency-care training; (3) simulation or virtual-patient applications relevant to emergency care; (4) formative feedback or assessment; (5) documentation, handoff, discharge communication, or translation when treated as educational targets; and (6) governance issues affecting the educational use of LLMs in emergency settings. Exclusion criteria were as follows: (1) conference abstracts without sufficient methodological detail; (2) duplicates; (3) purely technical benchmark studies without clear educational relevance; and (4) articles focused solely on routine clinical decision support, diagnostic prediction, triage automation, operational workflow, EHR documentation generation, model development, or technical benchmarking without a clear educational aim, learner or faculty population, training relevance, or implementation implication for emergency medicine education.

Two reviewers independently extracted data in duplicate from the included records. Extracted variables included original title, publication year, journal or source, DOI where available, LLM or AI system evaluated, participants or population when applicable, and educational context. Disagreements in extracted information or evidence classification were first resolved through discussion between the two reviewers. If consensus could not be reached, a third senior reviewer adjudicated. Given the heterogeneity of the included literature in study design, population, LLM system, educational context, comparator, and outcome reporting, quantitative synthesis was not appropriate. Many included records were exploratory, descriptive, benchmark-based, simulation-based, documentation-focused, or governance-oriented, and they did not report sufficiently comparable effect estimates or standardized educational outcomes for meta-analysis. Therefore, findings were summarized narratively rather than pooled statistically.

## Results

**Figure 1:**
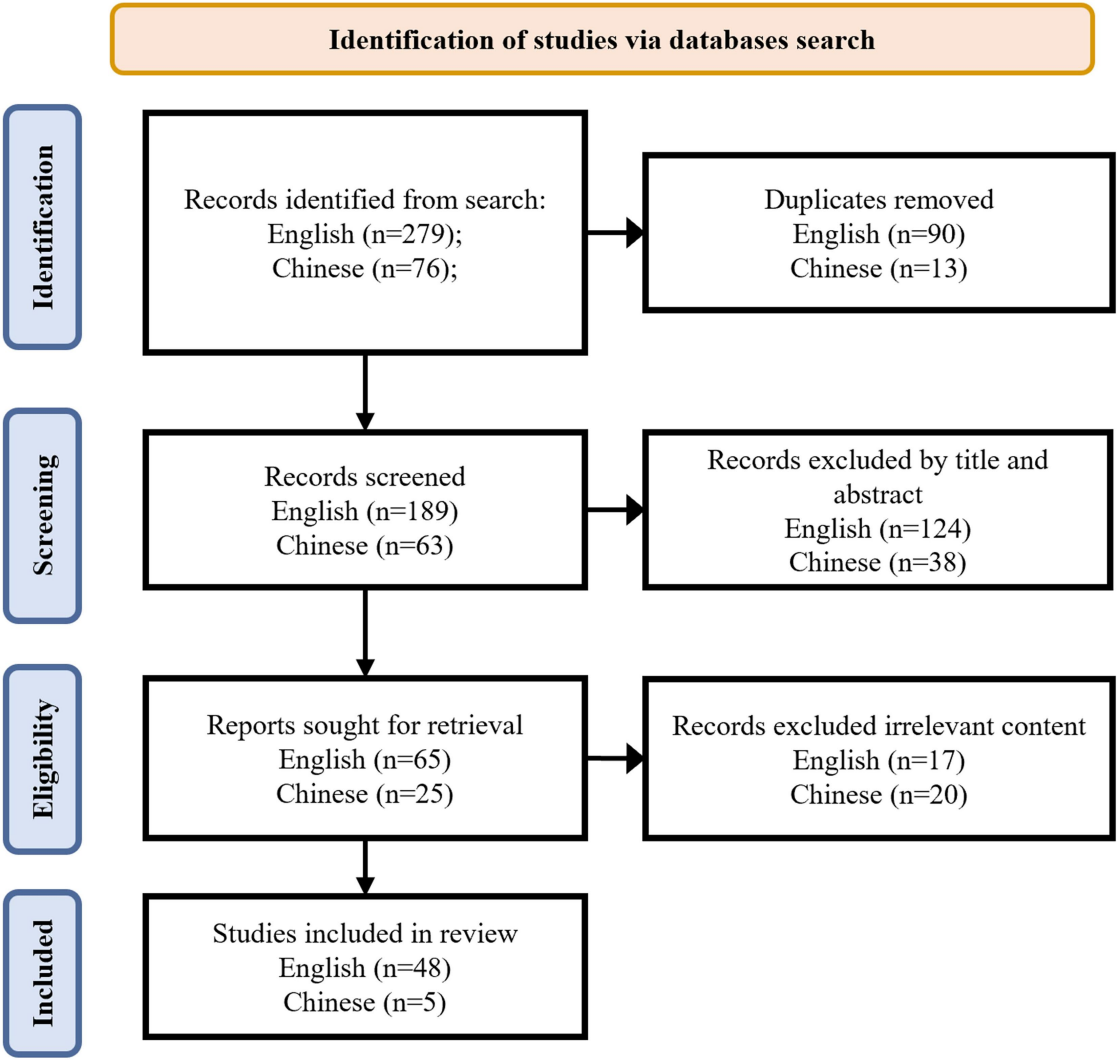
Identification of studies via database search.

The database search identified 279 English-language records and 76 Chinese-language records. After removing 90 English-language and 13 Chinese-language duplicates, 189 English-language and 63 Chinese-language records were screened. Full texts were sought for 65 English-language and 25 Chinese-language reports, and 48 English-language and 5 Chinese-language studies were ultimately included (Figure 1).

### Educational applications of LLMs in emergency medicine

The reviewed literature clustered into seven application domains, summarized in Table 1.

**Table 1:** Representative LLM-supported use cases in emergency medicine education.

Use case	Educational value in emergency medicine	Example outputs	Key references
Just-in-time tutoring and protocol translation	Clarifies differentials, pharmacology, resuscitation priorities, and disposition logic during or between shifts.	Protocol explainers, oral-board prompts, shift learning goals	(17–20)
Case and scenario generation	Expands rehearsal of low-frequency, high-consequence events that are difficult to guarantee in routine clinical exposure.	Sepsis, toxicology, airway, trauma, resuscitation, and mass-casualty micro-scenarios	(17, 19–21, 23–27, 47, 69)
Virtual patients and dialogic rehearsal	Supports history taking, family communication, and handoff rehearsal under diagnostic uncertainty.	Simulated patients, difficult-conversation scripts, iterative questioning	(22, 29–31, 47)
Feedback and formative assessment support	Drafts debrief notes and aligns comments with defined domains such as Mini-CEX or DOPS.	Debrief summaries, reflection prompts, rubric-aligned feedback	(32–35)
Documentation and discharge coaching	Improves note organization, patient-facing explanations, and transitions of care.	Discharge instructions, handoff outlines, plain-language explanations	(36, 43–48)
Research and educator workflow support	Reduces first-draft burden for reading lists, case banks, and educator-facing teaching materials.	Literature synthesis, case-bank drafting, debrief templates	(11, 17–20, 49, 65)
Examination and competency-assessment support	Supports item drafting, benchmark interpretation, rubric mapping, and competency-oriented feedback while requiring human validation.	Board-style questions, MCQ critique, CPR-skill scoring rubrics, explanation keys	(33, 34, 37–41)

DOPS, direct observation of procedural skills; LLM, large language model; Mini-CEX, mini-clinical evaluation exercise.

### Just-in-time tutoring and resource generation

In emergency medicine education, just-in-time tutoring and rapid resource generation were among the most practical LLM-supported uses identified in the reviewed literature (17–20). Teaching in the emergency department often occurs in brief, interrupted intervals, which limits opportunities for extended bedside explanation or structured small-group instruction (1, 17). Under these conditions, LLMs can generate learner-facing materials, including concise summaries, chief-complaint-based teaching notes, oral-board prompts, protocol explainers, flashcards, and shift-ready problem sets (20). Prompt-engineering and emergency-focused studies suggest that structured prompts can improve the relevance and consistency of AI-generated learning resources, although outputs still require clinical review (19, 20). These functions are most useful when they translate local workflows, triage priorities, and common emergency presentations into concise teaching tools that can be used during or between shifts (17, 19). Their value lies not only in saving preparation time, but also in making teaching more responsive to the pace, variability, and cognitive load of emergency care (18).

### Case generation, simulation, and virtual patients

Case generation, simulation, and virtual-patient applications were prominent LLM-supported uses in emergency medicine education (21, 22). Emergency training requires repeated exposure to high-acuity but relatively infrequent scenarios, such as airway emergencies, toxicologic crises, sepsis, trauma resuscitation, sudden clinical deterioration, and cardiac arrest (21, 23, 24). Chinese emergency-training literature also emphasized virtual reality, high-fidelity simulation, domain-specific virtual systems, and LLM- or DeepSeek-related opportunities for emergency residency training (25–28). LLM-supported case generation can address uneven exposure by producing adaptable scenarios, interactive patient dialogues, AI team-member simulations, and structured debrief prompts tailored to emergency presentations and learner level (20, 21). Pilot, qualitative, and proof-of-concept studies suggest that LLM-supported simulation may support team-leadership practice, clinical-scenario drafting, diagnostic rehearsal, and difficult-conversation training (21). Broader virtual-patient literature suggests that LLMs can expand opportunities for history-taking practice and complex multimorbidity scenarios, although performance varies across systems (29–31). In emergency medicine, the major advantage of these tools is not replacement of live simulation, but expansion of access to repeated, case-based rehearsal when faculty time, simulation resources, and case exposure are constrained (19, 23).

### Formative feedback and work-based assessment support

Formative feedback and work-based assessment support are highly relevant to emergency medicine education (32–34). In the emergency department, feedback is often delayed, brief, or inconsistently documented because supervisors must balance teaching with patient flow, interruptions, and urgent clinical decisions (2, 17). As a result, assessment tools such as Mini-CEX and DOPS can lose educational value when observations are not translated into timely and specific feedback (32). LLMs may help by organizing observed performance according to explicit domains, drafting debrief points, and converting fragmented comments into more actionable formative feedback (32, 33, 35, 36). Emerging work on AI-generated multiple-choice questions and AI-assisted CPR-skill evaluation suggests that LLMs may support competency-assessment workflows, but only when paired with psychometric review, expert calibration, and human oversight (33, 34). Accordingly, the most credible role of LLMs in this area is not autonomous evaluation, but support for clearer, more structured, and more timely formative assessment in the fast-paced emergency setting (33, 34).

### Examination and competency-assessment support

Examination studies provide an additional but more limited application area (37–41). Several emergency medicine studies have tested LLMs on board-style questions, residency or clerkship examinations, specialist examinations, and AI-generated multiple-choice items (33, 41). These studies are useful for understanding model knowledge, item-writing potential, and the need for psychometric validation, but high performance on selected examination questions or simulated skill-scoring tasks should not be interpreted as evidence of validity, reliability, fairness, accountability, or readiness for high-stakes competency judgment (33, 38, 42). In educational practice, the safer role is to use LLMs for supervised item drafting, question critique, explanation generation, rubric mapping, and formative feedback support, followed by expert calibration, learner-outcome validation, subgroup fairness testing, and clear error-review procedures (41).

### Documentation and communication coaching

Documentation and communication coaching represent another important use case in emergency medicine education (43, 44). In the emergency department, documentation does not merely record care; it also reflects diagnostic reasoning, risk communication, disposition planning, and transitions of care (43). Emergency-care workflow studies suggest that LLM-assisted discharge, handoff, and documentation tools may improve completeness, readability, or structured information transfer, but these findings should be interpreted as indirect evidence for educational implementation rather than direct evidence of educational effectiveness (45, 46). Related work on synthetic emergency dialogues and difficult-conversation training suggests that LLMs can support communication training, scenario design, and structured extraction of clinically relevant information from simulated encounters (47). For emergency medicine learners, such tools may be useful not only for improving written outputs, but also for practicing how to explain uncertainty, communicate return precautions, structure handoffs, and deliver patient-centered instructions under time pressure (43, 44, 46). However, multilingual discharge communication and machine translation remain safety-sensitive and require post-editing, local validation, and escalation pathways for uncertainty (48).

### Research and educator workflow support

LLMs may also support emergency medicine education indirectly by improving educator workflow and academic productivity (13, 49). Across the broader medical education literature, these tools have been used for literature summarization, manuscript drafting, curriculum design, and evolving faculty roles in digital teaching environments (11). In emergency medicine, such functions are valuable because educators often work in clinically demanding environments with limited protected teaching time (18, 50, 51). LLMs can assist with low-risk but time-consuming tasks such as drafting first-pass teaching cases, organizing debrief frameworks, summarizing evidence for case conferences, preparing flashcards or teaching prompts, and tailoring educational materials to common emergency presentations (17, 19). By reducing the burden of preliminary drafting and information synthesis, these tools may allow faculty to devote more time to contextual teaching, bedside supervision, and individualized learner support (49). However, their use remains most appropriate when they augment, rather than replace, educator judgment (11).

### Major challenges

**Figure 2:**
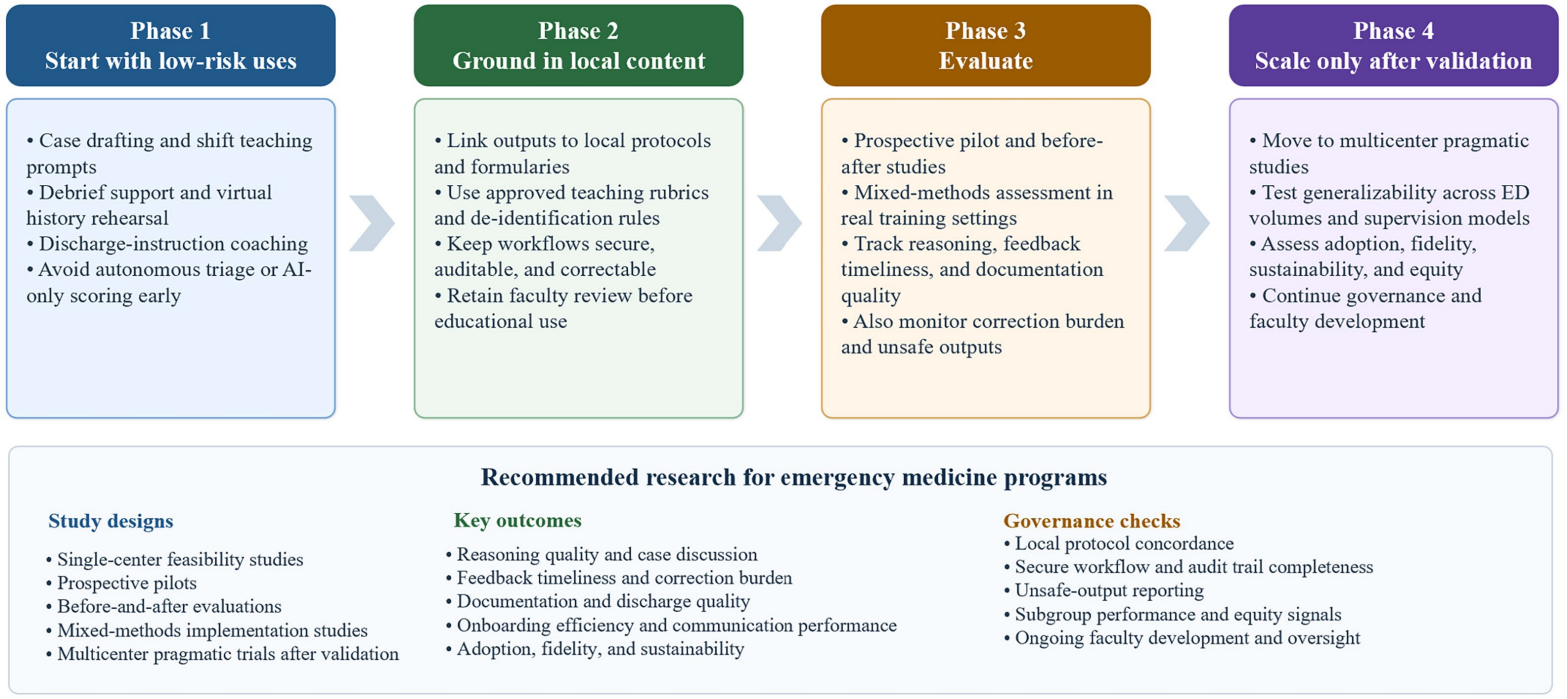
Phased pathway for safe adoption of LLM-enabled emergency medicine education.

Despite these opportunities, the reviewed literature repeatedly identified five risk domains that were decisive for responsible adoption: clinical grounding, safety and governance, educational effectiveness, workflow impact, and equity and faculty readiness (Table 2; Figure 2).

**Table 2:** Implementation priorities and evaluation domains for LLM-enabled emergency medicine education.

Domain	Key question	Illustrative actions or metrics	Key references
Clinical grounding	Does the tool reflect local emergency protocols, escalation pathways, and documentation norms?	Institution-approved knowledge sources, protocol-concordance audits, faculty review logs	(52, 54, 59, 60, 68, 70)
Safety and governance	Are privacy, access control, and unsafe-output reporting built into the workflow?	Secure environment, de-identification standards, audit logs, incident-reporting pathways	(45, 53, 59)
Educational effectiveness	Does use improve reasoning, communication, or procedural preparation rather than only satisfaction?	Simulation scores, Mini-CEX trends, note-quality rubrics, feedback timeliness	(32, 33, 35, 37)
Workflow impact	Does adoption reduce hidden rework or simply shift editing burden to faculty?	Onboarding duration, faculty preparation time, note-edit burden, discharge-writing turnaround	(45, 49)
Equity and faculty readiness	Does performance remain acceptable across languages, learner levels, and patient scenarios?	Multilingual testing, subgroup audits, faculty development completion, unsafe-output taxonomy	(60–62, 64)

### Hallucination and context mismatch

The most immediate challenge is hallucination, namely fluent but incomplete, fabricated, or locally inappropriate output (9, 52, 53). In emergency medicine, this problem has particular significance because decisions are often made under time pressure, with limited information and little tolerance for error (54–58). Even when an LLM response appears reasonable, it may conflict with local triage processes, institutional protocols, formulary restrictions, escalation pathways, or documentation expectations (52, 55, 58). This is especially concerning in scenarios such as chest pain triage, sepsis recognition, anticoagulation reversal, cardiac arrest, basic life support, or disposition planning, where small deviations from protocol can have important downstream consequences (54, 55, 58). Studies of triage-related LLM performance show substantial variability across models and settings, and resuscitation-focused studies show that publicly available chatbots may omit essential guideline details or produce potentially harmful advice (58). In emergency medicine education, the central issue is therefore not only whether a model can generate plausible answers, but whether those answers are contextually aligned with local emergency care (59, 60).

### Automation bias and erosion of diagnostic reasoning

A second major concern involves automation bias and the possible erosion of independent diagnostic reasoning (61–63). Emergency medicine training depends on the ability to generate differential diagnoses, prioritize life-threatening conditions, recognize clinical deterioration, and revise decisions as new information emerges (1, 2). These cognitive skills may be weakened if trainees treat LLMs as answer engines rather than tools for structured reflection or guided learning (61, 63). Viewpoint and survey-based papers warn that the convenience and fluency of model output may encourage premature acceptance, especially among less experienced learners (64). Examination studies in emergency medicine show that model performance can be high on selected board-style or multiple-choice tasks, but such benchmark performance does not establish safe clinical reasoning, educational validity, or readiness for autonomous assessment (37, 40). Experimental evidence highlights the same tension: structured LLM support can improve immediate diagnostic performance, yet convenience may also encourage overtrust and reduced independent hypothesis generation (63).

### Limits of relational authenticity and AI-assisted assessment

A further limitation concerns the relational nature of emergency care and the difficulty of capturing it through AI-generated interaction (29, 31). Emergency medicine is not solely a cognitive discipline; it also requires learners to communicate with frightened patients, distressed families, angry visitors, and multidisciplinary teams in moments of uncertainty, grief, or urgency (2). Although virtual patients and LLM-based conversational systems broaden opportunities for practice, they cannot fully reproduce the emotional intensity, nonverbal communication, ethical complexity, and interpersonal unpredictability of real bedside encounters (29, 30). Similar caution applies to assessment (34, 65). AI-generated comments may help organize feedback and make debriefing more efficient, but current evidence does not support stand-alone AI judgment for complex outcomes such as prioritization, professionalism, teamwork, or contextual decision making (32, 34). LLMs may assist with preparation and feedback structure, but they cannot substitute for human observation, interpretation, and relational teaching (49).

### Privacy, cybersecurity, and governance

Privacy, cybersecurity, and governance remain central barriers to safe implementation (52, 53, 59). Many educational use cases in emergency medicine depend on case narratives, notes, handoff content, discharge materials, or de-identified clinical records (45). Even de-identified emergency data may retain contextual details that create privacy concerns, especially in rare presentations, trauma cases, or small institutional settings (45). Emergency departments also rely on fast-moving, team-based communication workflows, which makes informal or unsecured use of AI tools particularly risky (45, 59). Secure deployment, role-based access control, auditability, and cyber-resilience are therefore prerequisites rather than optional enhancements (53, 59). Recent healthcare informatics and policy literature emphasizes that enterprise-level infrastructure, governance standards, and regulatory oversight are essential for responsible use of generative AI in clinical environments (52, 53). EMS documentation work further illustrates that integration into clinical documentation workflows requires careful attention to data security, workflow fit, and accountability (45).

For educational deployment, ethical governance of LLMs should explicitly address consent where applicable, data provenance, transparency, accountability, and patient safety when real or simulated emergency cases are used for teaching, feedback, or documentation coaching (52, 66, 67). Case narratives, handoff notes, discharge instructions, trauma scenarios, EMS reports, and rare presentations may remain potentially identifiable even after nominal de-identification, especially in small institutions, resource-limited settings, or crisis events (45, 52, 66). Therefore, LLM-enabled educational tools should preferably be deployed within secure institutional environments rather than through unsecured public interfaces, with role-based access control, data minimization, de-identification standards, audit logs, and output traceability (53, 59). Clear responsibility should be assigned for reviewing, approving, correcting, and reporting AI-generated outputs, because accountability can become ambiguous when model-generated content is incorporated into teaching materials, feedback, or documentation-related education (52, 53). These safeguards are necessary to support responsible deployment while reducing risks related to privacy breaches, unsafe outputs, automation bias, and patient-safety harm in emergency medicine education (52, 66).

### Bias, multilingual inequity, and implementation readiness

Model performance is not uniform across languages, learner levels, institutional resources, or sociocultural settings (60, 62). This issue is particularly salient in emergency care, where triage communication, discharge understanding, informed consent, and team interaction are shaped by language, culture, and local workflow (48). A model that performs well in English-language benchmark tasks may still perform poorly in multilingual emergency encounters, locally specific documentation settings, or communication with patients of varying literacy levels (46, 48). Studies of GPT-generated multilingual discharge instructions and machine translation of patient-specific discharge instructions show the promise of language support but also underscore the need for expert review and safety validation (48). Papers addressing healthcare deployment and educator roles suggest that rushed adoption, weak localization, and inadequate faculty preparation could widen rather than reduce educational inequities (49, 60). Implementation readiness therefore requires local adaptation, faculty development, institutional guidance, and continuous monitoring of how LLM use affects learning and communication in real emergency settings (46).

Equity considerations in LLM-supported emergency medicine education should extend beyond multilingual performance to include health literacy, disability access, rural and under-resourced settings, migrant populations, culturally diverse patients, and crisis-affected communities (46, 48, 60). LLM-generated educational or communication materials may be less safe or less effective when they assume high literacy, stable digital access, dominant-language communication, or culturally familiar examples (46, 48). These concerns are particularly important in emergency and disaster contexts, where patients and learners may face time pressure, displacement, language barriers, disability-related communication needs, or limited access to digital infrastructure (60). Therefore, evaluation of LLM-supported education should include subgroup testing across language, reading level, disability-related accessibility needs, learner experience, institutional resource setting, and culturally diverse or crisis-affected populations (46, 60). Without such stratified evaluation, LLM-supported emergency medicine education could unintentionally reinforce existing disparities in communication quality, preparedness training, and access to safe educational support (48, 60).

### Implementation pathways for responsible adoption

Based on the reviewed literature and synthesis, we propose a phased implementation strategy rather than broad, unsupervised deployment (16, 53, 59). In this framework, faculty-reviewed case drafting, debrief prompts, virtual history rehearsal, and teaching-material generation were categorized as low-risk uses; formative feedback drafting, simulation dialogue, handoff-note practice, documentation coaching, and discharge-instruction practice were categorized as moderate-risk uses; and AI-assisted competency assessment, triage-related education, protocol translation, or any safety-sensitive learner-facing tool without immediate faculty review were categorized as high-risk uses. Each implementation phase should be linked to phase-specific evaluation metrics (Figure 2). In Phase 1, low-risk faculty-reviewed uses should be assessed by output accuracy, unsafe-output frequency, faculty editing burden, learner acceptability, and time saved in preparing teaching materials. In Phase 2, local grounding should be evaluated by protocol-concordance rates, compliance with institutional documentation formats, completeness of audit trails, privacy or de-identification incidents, and faculty approval rates before educational use. In Phase 3, pilot evaluation should measure feedback timeliness, learner reasoning quality, simulation or case-discussion performance, documentation and discharge-instruction quality, onboarding efficiency, communication performance, correction burden, and user acceptance. In Phase 4, scale-up should be permitted only after multicenter or multi-setting validation demonstrates generalizability, implementation fidelity, sustainability, subgroup equity across language and learner level, acceptable security performance, and continued faculty oversight.

In emergency medicine, early adoption should focus on low-risk, faculty-reviewed applications, such as case drafting, debrief prompts, virtual history rehearsal, handoff-note practice, discharge-instruction coaching, and educator-facing teaching materials, because these use cases are educationally valuable while remaining auditable and correctable (19, 36, 44). Future research should begin with prospective pilot studies, before-and-after studies, and mixed-methods evaluations in real emergency department training settings (35, 45, 46). These studies should examine whether LLM-supported tools improve feedback timeliness, reasoning support, documentation quality, onboarding efficiency, communication performance, and learner preparedness, while also measuring correction burden, unsafe outputs, protocol discordance, and user acceptance (21, 44, 46). After feasibility is established, multicenter pragmatic studies can test generalizability across emergency departments and training environments (59, 60). Throughout implementation, tools should remain grounded in local protocols, teaching rubrics, and secure workflows, with faculty oversight retained at every stage (52, 68).

### Future directions for AI in emergency medicine training

The future of AI in emergency medicine training will depend on whether these systems can deliver reliable, context-sensitive, and educationally valid support in the realities of emergency care (17, 60). Priority areas include accelerated onboarding of new trainees, scalable rehearsal of high-acuity and low-frequency scenarios, support for fragmented shift-based learning, more timely formative feedback, and greater consistency in teaching across instructors and clinical settings (17, 36, 46). AI tools should be aligned with local policies, documentation standards, and supervision structures rather than deployed as generic stand-alone systems (53, 61). Future research should move beyond feasibility and satisfaction outcomes to examine whether AI-supported training improves clinical reasoning, communication, handoff quality, documentation performance, resuscitation preparedness, and readiness for critical deterioration or surge events (21, 35, 45). Multilingual capability, privacy-preserving deployment, faculty readiness, and governance frameworks should remain central to implementation design (46, 48, 53).

## Limitations

This review had several limitations. First, it was a structured narrative review rather than a systematic review or meta-analysis, and the selection and synthesis of evidence therefore remained partly interpretive. Second, the current literature on LLMs in emergency medicine education is heterogeneous, with many studies being descriptive, exploratory, or based on benchmark performance rather than validated educational or patient-centered outcomes. Third, because this field is evolving rapidly, model-specific evidence, available tools, and implementation assumptions may change during manuscript preparation and peer review. Fourth, although both English-language and Chinese-language literature were included, relevant studies from other databases, gray literature, preprints, or unpublished institutional experiences may not have been captured. In addition, disaster medicine was not included as a primary search concept or eligibility focus, so disaster-specific LLM educational applications, such as surge response, mass-casualty preparedness, crisis communication, and health-system resilience training, may be underrepresented. Finally, this review did not perform formal risk-of-bias assessment or quantitative pooling, so its findings should be interpreted primarily as a thematic synthesis intended to inform future research and implementation.

## Conclusion

Large language models show meaningful potential in emergency medicine education, particularly for just-in-time teaching, case generation, formative feedback, assessment support, documentation coaching, communication rehearsal, and educator workflow support. Their value is especially relevant in emergency settings, where training is shaped by time pressure, uneven case exposure, and limited opportunities for individualized supervision. However, hallucination, context mismatch, automation bias, uncertain validity of AI-assisted assessment, privacy risk, multilingual inequity, and governance concerns remain major barriers to safe adoption. The available heterogeneous evidence favors cautious, phased, locally grounded, and faculty-supervised implementation rather than broad unsupervised use. Overall, LLMs should be viewed as supportive educational tools that may strengthen emergency medicine training when integrated with clear safeguards and sustained human oversight.

## References

[1] Townend W Long J Munro-Davies L Beet E. How do we educate the next generation of emergency physicians: Rcem 50. Emerg Med J. (2018) 35:159–61. doi: 10.1136/emermed-2017-207223, 29463636

[2] Al-Azri N. How to think like an emergency care provider: a conceptual mental model for decision making in emergency care. Int J Emerg Med. (2020) 13:17. doi: 10.1186/s12245-020-00274-0, 32299358 PMC7164351

[3] Wang H Fu T Du Y Gao W Huang K Liu Z . Scientific discovery in the age of artificial intelligence. Nature. (2023) 620:47–60. doi: 10.1038/s41586-023-06221-2, 37532811

[4] Thirunavukarasu A Ting D Elangovan K Gutierrez L Tan T Ting D. Large language models in medicine. Nat Med. (2023) 29:1930–40. doi: 10.1038/s41591-023-02448-8, 37460753

[5] Lee J Wu A Li D Kulasegaram K. Artificial intelligence in undergraduate medical education: a scoping review. Acad Med. (2021) 96:S62–s70. doi: 10.1097/acm.0000000000004291, 34348374

[6] Singhal K Azizi S Tu T Mahdavi S Wei J Chung H . Large language models encode clinical knowledge. Nature. (2023) 620:172–80. doi: 10.1038/s41586-023-06291-2, 37438534 PMC10396962

[7] Singhal K Tu T Gottweis J Sayres R Wulczyn E Amin M . Toward expert-level medical question answering with large language models. Nat Med. (2025) 31:943–50. doi: 10.1038/s41591-024-03423-7, 39779926 PMC11922739

[8] Sallam M. Chatgpt utility in healthcare education, research, and practice: systematic review on the promising perspectives and valid concerns. Healthcare (Basel). (2023) 11:887. doi: 10.3390/healthcare11060887, 36981544 PMC10048148

[9] Moor M Banerjee O Abad Z Krumholz H Leskovec J Topol E . Foundation models for generalist medical artificial intelligence. Nature. (2023) 616:259–65. doi: 10.1038/s41586-023-05881-4, 37045921

[10] Xu X Chen Y Miao J. Opportunities, challenges, and future directions of large language models, including Chatgpt in medical education: a systematic scoping review. J Educ Eval Health Prof. (2024) 21:6. doi: 10.3352/jeehp.2024.21.6, 38486402 PMC11035906

[11] Li R Wu T. Delving into the practical applications and pitfalls of large language models in medical education: narrative review. Adv Med Educ Pract. (2025) 16:625–36. doi: 10.2147/amep.s497020, 40271151 PMC12015179

[12] Mueller B Kinoshita T Peebles A Graber M Lee S. Artificial intelligence and machine learning in emergency medicine: a narrative review. Acute Med Surg. (2022) 9:e740. doi: 10.1002/ams2.740, 35251669 PMC8887797

[13] Kirubarajan A Taher A Khan S Masood S. Artificial intelligence in emergency medicine: a scoping review. J Am Coll Emerg Physicians Open. (2020) 1:1691–702. doi: 10.1002/emp2.12277, 33392578 PMC7771825

[14] Hwai H Ho Y Wang C Huang C. Large language model application in emergency medicine and critical care. J Formos Med Assoc. (2025) 124:696–9. doi: 10.1016/j.jfma.2024.08.032, 39198112

[15] Meyer N Meyer J. A practical guide to the utilization of Chatgpt in the emergency department: a systematic review of current applications, future directions, and limitations. Cureus. (2025) 17:e81802. doi: 10.7759/cureus.81802, 40330395 PMC12054779

[16] Preiksaitis C Ashenburg N Bunney G Chu A Kabeer R Riley F . The role of large language models in transforming emergency medicine: scoping review. JMIR Med Inform. (2024) 12:e53787. doi: 10.2196/53787, 38728687 PMC11127144

[17] Shaw J Mand S Williams S Cico S Bailes C Alvarez A . Practical applications of generative artificial intelligence for clinical teaching in the emergency department. AEM Educ Train. (2026) 10:e70146. doi: 10.1002/aet2.70146

[18] Basnawi A Koshak A. Application of artificial intelligence in advanced training and education of emergency medicine doctors: a narrative review. Emerg Care Med. (2024) 1:247–59. doi: 10.3390/ecm1030026

[19] Meynhardt C Meybohm P Kranke P Hölzing C. Advanced prompt engineering in emergency medicine and anesthesia: enhancing simulation-based e-learning. Electronics. (2025) 14:1028. doi: 10.3390/electronics14051028

[20] Ghaffari F Langarizadeh M Nabovati E Sabery M. Effectiveness of Chatgpt for clinical scenario generation: a qualitative study. Arch Acad Emerg Med. (2025) 13:e49. doi: 10.22037/aaemj.v13i1.2690, 40487903 PMC12145122

[21] Kanbakan A Berikol G İlhan B Altıntaş E Doğanay F. Artificial intelligence based resuscitation simulation: a pilot study of a novel approach to team leadership training. BMC Med Educ. (2026) 26:183. doi: 10.1186/s12909-025-08539-z, 41486153 PMC12870186

[22] Webb JJ. Proof of concept: using Chatgpt to teach emergency physicians how to break bad news. Cureus. (2023) 15:e38755. doi: 10.7759/cureus.38755, 37303324 PMC10250131

[23] Pasquariello L Sims J O'brien J Upperman J. Current and future applications of AI in EMS training: a scoping review. Cureus. (2025) 17:e90469. doi: 10.7759/cureus.90469, 40979032 PMC12445063

[24] Komasawa N Yokohira M. Simulation-based education in the artificial intelligence era. Cureus. (2023) 15:e40940. doi: 10.7759/cureus.40940, 37496549 PMC10368461

[25] Feng J Shao C Chen S Zhang Q Yao X Zheng S . Application of virtual reality technology in adult CPR training: a scoping review. Chin J Crit Care Med. (2024) 44:68–72. doi: 10.3969/j.issn.1002-1949.2024.01.012

[26] Chen J He X Zhang J. Opportunities and challenges brought by natural language large models represented by DeepSeek to standardized residency training in emergency medicine. Chin J Emerg Med. (2025) 34:616–9. doi: 10.3760/cma.j.issn.1671-0282.2025.05.002

[27] He X Luo H Tan J Huang Y Jiang H Wang M. Research progress of high-fidelity simulation teaching in clinical emergency nursing. Cap Food Med. (2025) 32:11–4. doi: 10.3969/j.issn.1005-8257.2025.15.006

[28] Wu H Zeng Y Tian Y. Research progress overview and prospects of post-disaster psychological first aid training based on virtual simulation technology. West China Med J. (2024) 39:1770–7. doi: 10.7507/1002-0179.202404213

[29] Abou KG. Revolutionizing medical education: Chatgpt3.5 ability to behave as a virtual patient. Med Sci Educ. (2024) 34:1559–64. doi: 10.1007/s40670-024-02121-w, 39758479 PMC11699164

[30] Zeng J Qi W Shen S Liu X Li S Wang B . Embracing the future of medical education with large language model-based virtual patients: scoping review. J Med Internet Res. (2025) 27:e79091. doi: 10.2196/79091, 41232097 PMC12661241

[31] Li D Lebai Lutfi S. Large language model-based virtual patient systems for history-taking in medical education: comprehensive systematic review. JMIR Med Inform. (2026) 14:e79039. doi: 10.2196/79039 41481915 PMC12811743

[32] Wang Y Chen G Chen J Jiang F. Application of Mini-Cex combined with Dops in standardized training of community outpatient residents. BMC Med Educ. (2024) 24:780. doi: 10.1186/s12909-024-05739-x, 39030518 PMC11264813

[33] Kaya M Sonmez E Halici A Yildirim H Coskun A. Comparison of Ai-generated and clinician-designed multiple-choice questions in emergency medicine exam: a psychometric analysis. BMC Med Educ. (2025) 25:949. doi: 10.1186/s12909-025-07528-6, 40597998 PMC12210704

[34] Wang L Mao Y Wang L Sun Y Song J Zhang Y. Suitability of Gpt-4o as an evaluator of cardiopulmonary resuscitation skills examinations. Resuscitation. (2024) 204:110404. doi: 10.1016/j.resuscitation.2024.110404, 39343124

[35] Brügge E Ricchizzi S Arenbeck M Keller M Schur L Stummer W . Large language models improve clinical decision making of medical students through patient simulation and structured feedback: a randomized controlled trial. BMC Med Educ. (2024) 24:1391. doi: 10.1186/s12909-024-06399-7, 39609823 PMC11605890

[36] Lee S Song J You S Kim J. Shifts in emergency physicians' attitudes toward large language model-based documentation: a pre- and post-implementation study. Sci Rep. (2025) 15:40643. doi: 10.1038/s41598-025-24659-4, 41285993 PMC12644546

[37] Pastrak M Kajitani S Goodings A Drewek A Lafree A Murphy A. Evaluation of Chatgpt performance on emergency medicine board examination questions: observational study. JMIR AI. (2025) 4:e67696–6. doi: 10.2196/67696, 40611478 PMC12231519

[38] Almehairi N Clark G Davis S. How well does Gpt-4 perform on an emergency medicine board exam? A comparative assessment. Can J Emerg Med. (2025) 27:969–73. doi: 10.1007/s43678-025-00951-0, 40610779

[39] Liu C Ho C Wu T. Custom GPTs enhancing performance and evidence compared with GPT-3.5, GPT-4, and GPT-4o? A study on the emergency medicine specialist examination. Healthcare (Basel). (2024) 12:1726. doi: 10.3390/healthcare12171726, 39273750 PMC11394718

[40] Al-Thani S Anjum S Bhutta Z Bashir S Majeed M Khan A . Comparative performance of ChatGPT, Gemini, and final-year emergency medicine clerkship students in answering multiple-choice questions: implications for the use of AI in medical education. Int J Emerg Med. (2025) 18:146. doi: 10.1186/s12245-025-00949-6, 40775272 PMC12329864

[41] Law A So J Lui C Choi Y Cheung K Kei-Ching Hung K . AI versus human-generated multiple-choice questions for medical education: a cohort study in a high-stakes examination. BMC Med Educ. (2025) 25:208. doi: 10.1186/s12909-025-06796-6, 39923067 PMC11806894

[42] Aykut A Yildirim C Gunsoy E. Large language models in emergency medicine: a critical appraisal of validity, reproducibility, and clinical utility (2020-2025). Eurasian J Emerg Med. (2026) 25:117–24. doi: 10.4274/eajem.galenos.2025.28412

[43] Song J Park J Kim J You S. Large language model assistant for emergency department discharge documentation. JAMA Netw Open. (2025) 8:e2538427. doi: 10.1001/jamanetworkopen.2025.38427, 41118162 PMC12541540

[44] Hartman V Zhang X Poddar R Mccarty M Fortenko A Sholle E . Developing and evaluating large language model-generated emergency medicine handoff notes. JAMA Netw Open. (2024) 7:e2448723. doi: 10.1001/jamanetworkopen.2024.48723, 39625719 PMC11615705

[45] Bai E Luo X Zhang Z Adelgais K Ali H Finkelstein J . Assessment and integration of large language models for automated electronic health record documentation in emergency medical services. J Med Syst. (2025) 49:65. doi: 10.1007/s10916-025-02197-w, 40381087

[46] Gimeno A Krause K D'souza S Walsh C. Completeness and readability of Gpt-4-generated multilingual discharge instructions in the pediatric emergency department. JAMIA Open. (2024) 7:ooae050. doi: 10.1093/jamiaopen/ooae050, 38957592 PMC11216721

[47] Moser D Bender M Sariyar M. Generating synthetic healthcare dialogues in emergency medicine using large language models. Stud Health Technol Inform. (2024) 321:235–9. doi: 10.3233/shti241099, 39575815

[48] Kong M Fernandez A Bains J Milisavljevic A Brooks K Shanmugam A . Evaluation of the accuracy and safety of machine translation of patient-specific discharge instructions: a comparative analysis. BMJ Qual Saf. (2026) 35:150–8. doi: 10.1136/bmjqs-2024-018384, 40633961 PMC12252260

[49] Li Z Li F Fu Q Wang X Liu H Zhao Y . Large language models and medical education: a paradigm shift in educator roles. Smart Learn Environ. (2024) 11:26. doi: 10.1186/s40561-024-00313-w

[50] An K Zhang J Wang X She Y Li S Li S. Integration and innovation: medical and health consortia improving continuing medical education in China. Front Public Health. (2025) 13:1633363. doi: 10.3389/fpubh.2025.1633363, 41112659 PMC12528029

[51] An K Zhang R Zhu B Liu L Tang J Ma Y . Familiarity of teaching skills among general practitioners transfer training trainers in China: a cross-sectional survey. BMC Med Educ. (2023) 23:949. doi: 10.1186/s12909-023-04945-3, 38087271 PMC10717701

[52] Ong J Chang S William W Butte A Shah N Chew L . Ethical and regulatory challenges of large language models in medicine. Lancet Digit Health. (2024) 6:e428–32. doi: 10.1016/s2589-7500(24)00061-x, 38658283

[53] Meskó B Topol E. The imperative for regulatory oversight of large language models (or generative AI) in healthcare. NPJ Digit Med. (2023) 6:120. doi: 10.1038/s41746-023-00873-0, 37414860 PMC10326069

[54] Aqavil-Jahromi S Eftekhari M Akbari H Aligholi-Zahraie M. Evaluation of correctness and reliability of Gpt, bard, and Bing chatbots' responses in basic life support scenarios. Sci Rep. (2025) 15:11429. doi: 10.1038/s41598-024-82948-w, 40180985 PMC11968784

[55] Colakca C Ergın M Ozensoy H Sener A Guru S Ozhasenekler A. Emergency department triaging using ChatGPT based on emergency severity index principles: a cross-sectional study. Sci Rep. (2024) 14:22106. doi: 10.1038/s41598-024-73229-7, 39333599 PMC11436771

[56] Masanneck L Schmidt L Seifert A Kölsche T Huntemann N Jansen R . Triage performance across large language models, Chatgpt, and untrained doctors in emergency medicine: comparative study. J Med Internet Res. (2024) 26:e53297. doi: 10.2196/53297, 38875696 PMC11214027

[57] Pasli S Yadigaroğlu M Kirimli E Beşer M Unutmaz İ Ayhan A . ChatGPT-supported patient triage with voice commands in the emergency department: a prospective multicenter study. Am J Emerg Med. (2025) 94:63–70. doi: 10.1016/j.ajem.2025.04.040, 40273640

[58] Haim G Saban M Barash Y Cirulnik D Shaham A Eisenman B . Evaluating large language model-assisted emergency triage: a comparison of acuity assessments by GPT-4 and medical experts. J Clin Nurs. (2024) 1–7. doi: 10.1111/jocn.17490, 39610042 PMC13569248

[59] Ng M Helzer J Pfeffer M Seto T Hernandez-Boussard T. Development of secure infrastructure for advancing generative artificial intelligence research in healthcare at an academic medical center. J Am Med Inform Assoc. (2025) 32:586–8. doi: 10.1093/jamia/ocaf005, 39836496 PMC11833461

[60] Peng Y Malin B Rousseau J Wang Y Xu Z Xu X . From GPT to DeepSeek: significant gaps remain in realizing AI in healthcare. J Biomed Inform. (2025) 163:104791. doi: 10.1016/j.jbi.2025.104791, 39938624 PMC12188495

[61] Nguyen T. Chatgpt in medical education: a precursor for automation Bias? JMIR Med Educ. (2024) 10:e50174. doi: 10.2196/50174, 38231545 PMC10831594

[62] Wu Y Zheng Y Feng B Yang Y Kang K Zhao A. Embracing Chatgpt for medical education: exploring its impact on doctors and medical students. JMIR Med Educ. (2024) 10:e52483. doi: 10.2196/52483, 38598263 PMC11043925

[63] Goh E Gallo R Hom J Strong E Weng Y Kerman H . Large language model influence on diagnostic reasoning: a randomized clinical trial. JAMA Netw Open. (2024) 7:e2440969. doi: 10.1001/jamanetworkopen.2024.40969, 39466245 PMC11519755

[64] Dsouza J. A student's viewpoint on ChatGPT use and automation bias in medical education. JMIR Med Educ. (2024) 10:e57696. doi: 10.2196/57696, 38623729 PMC11034419

[65] Ahsan Z. Integrating artificial intelligence into medical education: a narrative systematic review of current applications, challenges, and future directions. BMC Med Educ. (2025) 25:1187. doi: 10.1186/s12909-025-07744-0, 40849650 PMC12374307

[66] Zhui L Fenghe L Xuehu W Qining F Wei R. Ethical considerations and fundamental principles of large language models in medical education: viewpoint. J Med Internet Res. (2024) 26:e60083. doi: 10.2196/60083, 38971715 PMC11327620

[67] Elbattah M Arnaud E Ghazali Da Dequen G Exploring the ethical challenges of large language models in emergency medicine: a comparative international review 2024 IEEE International Conference on Bioinformatics and Biomedicine (Bibm): 3–6 Dec. 2024. Piscataway, NJ, USA. (2024) 5750–5755.

[68] Pham C Govender R Tehami S Chavez S Adepoju O Liaw W. Chatgpt's performance in cardiac arrest and bradycardia simulations using the American Heart Association's advanced cardiovascular life support guidelines: exploratory study. J Med Internet Res. (2024) 26:e55037. doi: 10.2196/55037, 38648098 PMC11074885

[69] An G Zhang Q Wu H Yu W. Application of a virtual simulation system for chemical defense treatment in chemical defense training. China J Emerg Resusc Disaster Med. (2026) 21:413–416, 434. doi: 10.3969/j.issn.1673-6966.2026.03.026

[70] Birkun A Gautam A. Large language model (LLM)-powered chatbots fail to generate guideline-consistent content on resuscitation and may provide potentially harmful advice. Prehosp Disaster Med. (2023) 38:757–63. doi: 10.1017/s1049023x23006568, 37927093

