# Supplementary material for: Large language models in emergency medicine education: opportunities, challenges, and implementation pathways
Source: Front Public Health. 2026 Aug 5;14:1899528. doi: 10.3389/fpubh.2026.1899528 (PMC13485943; doi:10.3389/fpubh.2026.1899528)
Supplement: Supplementary file 1 [file Data_Sheet_1.PDF]

Supplementary Appendix S1. Search Strategy and Included-Study Evidence Map

Note. This appendix was prepared to strengthen methodological transparency and disclose the 48 English-language and 5 Chinese-language included records. Study type, educational context, outcome/validation focus, and evidence-status fields were standardized for the revised narrative synthesis. The table is intended to support transparent evidence mapping and should be reconciled with the final full-text extraction file during author proofing.

A. Supplementary Table S1. Review type and reporting alignment

Review type: structured narrative review informed by SANRA principles. The manuscript has been revised to clarify that the review was not a PRISMA-style systematic review or meta-analysis. The transparent flow diagram, database-specific search syntax, screening steps, extraction matrix, and evidence mapping were retained to improve reproducibility.

B. Supplementary Table S2. Search dates, databases, and language handling

Item	Specification
Databases	PubMed, Web of Science Core Collection, CNKI, and Wanfang Data.
Search date	10 April 2026.
Search period	1 January 2023 to 10 April 2026.
Language handling	English-language records were searched in PubMed and Web of Science; Chinese-language records were searched in CNKI and Wanfang using equivalent Chinese terms.
Records included	48 English-language records and 5 Chinese-language records.

C. Supplementary Table S3. Database-specific search strategies (PubMed example)

Step	PubMed syntax
**#1 LLM/generative AI block**	"Large Language Models"[MeSH Terms] OR "Generative Artificial Intelligence"[MeSH Terms] OR "large language model*"[Title/Abstract] OR "generative AI"[Title/Abstract] OR ChatGPT[Title/Abstract] OR GPT[Title/Abstract]
**#2 Emergency block**	Emergency Medicine"[MeSH Terms] OR "Emergency Service, Hospital"[MeSH Terms] OR "Resuscitation"[MeSH Terms] OR "emergency medicine"[Title/Abstract] OR "emergency department"[Title/Abstract] OR resuscitation[Title/Abstract]
**#3 Education block**	"Education"[MeSH Terms] OR "Education, Medical"[MeSH Terms] OR "Teaching"[MeSH Terms] OR education[Title/Abstract] OR training[Title/Abstract] OR teaching[Title/Abstract]
**#4 Combined search**	#1 AND #2 AND #3
**#5 Date-limited search**	#4 AND ("2023/01/01"[Date - Publication] : "2026/04/10"[Date - Publication])

D. Supplementary Table S4. Eligibility, screening, extraction, and evidence classification

Component	Revised specification
Inclusion criteria	Records addressing emergency medicine or EMS education; resuscitation or emergency-care training; simulation or virtual-patient applications relevant to emergency or disaster care; formative feedback or assessment; documentation, handoff, discharge communication, translation, or communication rehearsal as educational targets; or governance, ethics, privacy, and implementation issues directly affecting educational use of LLMs in emergency settings.
Exclusion criteria	Conference abstracts without sufficient methodological detail; purely technical benchmarks without educational or emergency-care relevance; articles focused only on routine clinical decision support without educational implications; non-emergency contexts without transferable training relevance; and duplicates.
Screening	Two reviewers independently screened titles/abstracts and then full texts; disagreements were resolved by discussion and consensus.
Extraction variables	Author/year, country or setting when available, design, learner/professional population, LLM or AI system, comparator, educational or implementation context, outcomes, validation approach, principal limitations, and directness for emergency medicine education.
Evidence classification	Direct emergency medicine education evidence; indirect emergency-care workflow/communication evidence; broader medical-education/governance evidence. Claims in the revised synthesis were limited according to these categories.

E. Supplementary Table S5. Included records

ID	Original title / Excel title	Year	Journal / Source	DOI	Study design / evidence type	Country / Setting	Outcomes / validation focus
E1	A comparative analysis of the performance of large language models in the basic life support exam: Comprehensive evaluation of ChatGPT-4o, Gemini 2.0, Claude 3.5, and DeepSeek R1	2025	Annals of Clinical and Analytical Medicine	10.4328/ACAM.22758	Comparative LLM benchmark / exam-performance evaluation; direct resuscitation-education evidence	Türkiye / BLS examination benchmark*	Model accuracy and comparative performance on BLS examination items; guideline-consistent resuscitation knowledge
E2	Advanced Prompt Engineering in Emergency Medicine and Anesthesia: Enhancing Simulation-Based e-Learning	2025	Electronics	10.3390/electronics14051028	Prompt-engineering and simulation-based e-learning design/evaluation; direct EM/anesthesia simulation-education evidence	Germany	Quality and feasibility of AI-supported prompts/scenarios for simulation-based e-learning
E3	AI performance in emergency medicine fellowship examination: comparative analysis of ChatGPT-4o, Gemini 2.0, Claude 3.5, and DeepSeek R1 models	2025	Turkish Journal of Medical Sciences	10.55730/1300-0144.6083	Comparative LLM benchmark / examination-performance evaluation; direct EM education evidence	Türkiye	Accuracy and comparative model performance on emergency medicine fellowship examination questions
E4	AI versus human-generated multiple-choice questions for medical education: a cohort study in a high-stakes examination	2025	BMC Med Educ	10.1186/s12909-025-06796-6	Cohort study; AI-vs-human MCQ generation/evaluation; broader medical-education evidence	Hong Kong, China	Question quality, examination performance, and validity/psychometric focus for high-stakes assessment
E5	An AI-Driven Trainee Performance Evaluation in XR-Based CPR Training System for Enhancing Personalized Proficiency	2026	Electronics	10.3390/electronics15020376	AI/XR system development and trainee-performance validation; direct CPR education evidence	South Korea	Trainee CPR proficiency, performance metrics, and personalized feedback/validation of the AI-driven evaluation system
E6	Artificial intelligence and informatics in neonatal resuscitation	2024	Seminars in Perinatology	10.1016/j.semperi.2024.151992	Narrative review / informatics perspective; neonatal resuscitation education and implementation evidence	United States / neonatal resuscitation informatics context*	AI and informatics applications for neonatal resuscitation; implications for education, training, and quality improvement
E7	Artificial intelligence based resuscitation simulation: a pilot study of a novel approach to team leadership training	2026	BMC Med Educ	10.1186/s12909-025-08539-z	Pilot simulation study; direct resuscitation team-leadership education evidence	Türkiye	Feasibility and educational effect of AI-based resuscitation simulation for team leadership training
E8	Assessment and Integration of Large Language Models for Automated Electronic Health Record Documentation in Emergency Medical Services	2025	J Med Syst	10.1007/s10916-025-02197-w	Assessment and integration study; indirect EMS documentation/workflow education evidence	United States / EMS documentation context*	Documentation quality, completeness, integration feasibility, and potential use for documentation coaching
E9	AutoCLC: Towards Automated Assessment and Feedback on Closed-Loop Communication in Team-based Healthcare Simulation Training	2026	ACM Transactions on Computing for Healthcare	10.1145/3768164	Automated assessment/feedback model development; direct team-simulation communication-training evidence	Not country-specific / simulation-training dataset; full-text verification required	Detection, assessment, and feedback on closed-loop communication in team-based simulation training
E10	Automatic Generation of Learning Material for Residential Students in ED: Prompt Engineering and Evaluation	2024	2024 21st International Conference on Information Technology Based	10.1109/ITHET61869.2024.10837651	Prompt-engineering and learning-material generation evaluation; direct ED learner/resident	Italy / ED resident learning-material generation*	Quality, relevance, and usability of automatically generated ED learning materials

			Higher Education and Training (ITHET)		education evidence		
E11	Beyond the chain of survival: a scoping review of artificial intelligence applications in cardiac arrest	2026	World Journal of Emergency Medicine	10.5847/wjem.j.1920-8642.2026.025	Scoping review; cardiac arrest and resuscitation AI evidence synthesis	Not country-specific / scoping review	Mapping of AI applications across the chain of survival, including training and education-relevant applications
E12	Can DALL-E 3 Reliably Generate 12-Lead ECGs and Teaching Illustrations?	2024	Cureus	10.7759/cureus.52748	Image-generation reliability evaluation; acute-care teaching-material evidence	Not country-specific / ECG teaching-image generation benchmark	Reliability and educational suitability of AI-generated 12-lead ECGs and teaching illustrations
E13	ChatG-PD? Comparing large language model artificial intelligence and faculty rankings of the competitiveness of standardized letters of evaluation	2024	AEM Education and Training	10.1002/aet2.11052	Comparative ranking/assessment study; EM application-evaluation evidence	United States / EM residency application-SLOE context	Agreement and competitiveness ranking validity for standardized letters of evaluation
E14	ChatGPT for use in technology-enhanced learning in anesthesiology and emergency medicine and potential clinical application of AI language models. Between hype and reality around artificial intelligence in medical use	2024	Die Anaesthesiologie / Anaesthesiologie	10.1007/s00101-024-01403-7	Narrative review/commentary; technology-enhanced learning and clinical application evidence	Germany	Potential educational uses, limitations, and implementation considerations for ChatGPT/AI language models
E15	ChatGPT's Performance in Cardiac Arrest and Bradycardia Simulations Using the American Heart Association's Advanced Cardiovascular Life Support Guidelines: Exploratory Study	2024	Journal of medical Internet research	10.2196/55037	Exploratory simulation performance study; direct ACLS/resuscitation education evidence	United States / AHA ACLS guideline context	Accuracy, safety, and guideline consistency in cardiac arrest and bradycardia simulation scenarios
E16	Comparative Analysis of Artificial Intelligence-Generated and Human-Written Personal Statements in Emergency Medicine Applications	2025	Cureus	10.7759/cureus.88818	Comparative content/assessment study; EM application-writing evidence	United States / EM application materials	Distinguishability, quality, and implications of AI-generated application materials
E17	Comparative performance of ChatGPT, Gemini, and final-year emergency medicine clerkship students in answering multiple-choice questions: implications for the use of AI in medical education	2025	International journal of emergency medicine	10.1186/s12245-025-00949-6	Comparative examination-performance study; direct EM clerkship education evidence	Qatar	MCQ accuracy and comparative performance; implications for AI use in medical education
E18	Comparison of AI-generated and clinician-designed multiple-choice questions in emergency medicine exam: a psychometric analysis	2025	BMC Med Educ	10.1186/s12909-025-07528-6	Psychometric comparative study; direct EM assessment evidence	Türkiye	Psychometric quality, difficulty, discrimination, and assessment suitability of AI-generated questions
E19	Comparison of performance of artificial intelligence tools in answering emergency medicine question pool: ChatGPT 4.0, Google Gemini and Microsoft Copilot	2025	Pakistan Journal of Medical Sciences	10.12669/pjms.41.4.11178	Comparative AI benchmark; direct EM question-pool assessment evidence	Türkiye	Accuracy and comparative tool performance on emergency medicine question pool items
E20	Completeness and readability of GPT-4-generated multilingual discharge instructions in the pediatric emergency department	2024	JAMIA open	10.1093/jamiaopen/ooae050	Evaluation of generated discharge instructions; indirect pediatric ED communication-education evidence	United States / pediatric ED discharge-instruction context	Completeness, readability, and language-related quality of patient-facing discharge instructions
E21	Conversational AI in tactical combat casualty care: Baseline GPT-4o improves medic decision-making	2025	Clinical Simulation in Nursing	10.1016/j.ecns.2025.101803	Simulation/decision-support experiment; EMS/tactical care training evidence	Czech Republic / tactical combat casualty care medics	Decision-making accuracy, speed, or scenario performance in tactical combat casualty care
E22	Custom GPTs Enhancing Performance and Evidence Compared with GPT-3.5, GPT-4, and GPT-4o? A Study on the Emergency Medicine Specialist Examination	2024	Healthcare (Basel, Switzerland)	10.3390/healthcare12171726	Comparative examination-performance study; direct EM specialist-examination evidence	Taiwan, China / emergency medicine specialist examination	Exam accuracy, evidence support, and comparative performance of customized versus general models

E23	Developing and Evaluating Large Language Model-Generated Emergency Medicine Handoff Notes	2024	JAMA Netw Open	10.1001/jamanetworkopen.2024.48723	Development and evaluation study; indirect EM handoff/documentation education evidence	United States / EM handoff-note context*	Quality, completeness, safety, and educational utility of LLM-generated handoff notes
E24	Differential performance of large language models in advanced cardiac life support assessment: A comprehensive multi-dimensional analysis of accuracy, consistency, and visual recognition capabilities	2026	PLOS ONE	10.1371/journal.pone.0347611	Multi-dimensional LLM benchmark; direct ACLS assessment evidence	Türkiye / ACLS assessment benchmark*	Accuracy, consistency, and visual-recognition capability in ACLS assessment
E25	Effectiveness of ChatGPT for Clinical Scenario Generation: A Qualitative Study	2025	Archives of academic emergency medicine	10.22037/aaemj.v13i1.2690	Qualitative study; clinical-scenario generation education evidence	Iran / clinical scenario generation*	Perceived effectiveness, quality, and usefulness of ChatGPT-generated clinical scenarios
E26	Embracing Large Language Models for Adult Life Support Learning	2024	Cureus	10.7759/cureus.75961	Narrative educational perspective/commentary; adult life support learning evidence	United Kingdom / Resuscitation Council UK adult life support learning	Opportunities, limitations, and implementation considerations for LLMs in adult life support learning
E27	Empowering Women in Academic Emergency Medicine: A Time-Efficient Workshop Incorporating Large Language Models to Enhance Leadership Skills	2026	AEM Education and Training	10.1002/aet2.70128	Educational workshop/curriculum report; faculty/leadership development evidence	United States / academic EM leadership workshop	Feasibility and perceived value of incorporating LLMs into leadership-skills training
E28	Evaluating Artificial Intelligence Competency in Education: Performance evaluation of ChatGPT in the neonatal resuscitation program exam	2025	Resuscitation	10.1016/j.resuscitation.2025.110563	Exam-performance evaluation; direct neonatal resuscitation education evidence	Türkiye / neonatal resuscitation program exam*	ChatGPT accuracy and reliability on neonatal resuscitation program examination content
E29	Evaluating the accuracy and reliability of AI chatbots in disseminating the content of current resuscitation guidelines: a comparative analysis between the ERC 2021 guidelines and both ChatGPTs 3.5 and 4	2024	Scandinavian Journal of Trauma, Resuscitation and Emergency Medicine	10.1186/s13049-024-01266-2	Comparative guideline-concordance evaluation; resuscitation education evidence	Germany / European Resuscitation Council guideline context	Accuracy and reliability of chatbot dissemination of current resuscitation guideline content
E30	Evaluation of a Large Language Model on the American Academy of Pediatrics' PREP Emergency Medicine Question Bank	2024	Pediatric Emergency Care	10.1097/PEC.0000000000003271	Question-bank performance evaluation; direct pediatric EM education evidence	United States / AAP PREP Emergency Medicine question bank	LLM accuracy and performance on pediatric emergency medicine question-bank items
E31	Evaluation of ChatGPT Performance on Emergency Medicine Board Examination Questions: Observational Study	2025	Jmir ai	10.2196/67696	Observational examination-performance study; direct EM assessment evidence	United States / emergency medicine board examination context	ChatGPT accuracy and performance on emergency medicine board examination questions
E32	Evaluation of ChatGPTs Performance in Türkiye's First Emergency Medicine Sub-Specialization Exam	2025	Eurasian Journal of Emergency Medicine	10.4274/eajem.galenos.2025.32644	Examination-performance evaluation; direct EM subspecialty assessment evidence	Türkiye	ChatGPT performance on Türkiye's emergency medicine sub-specialization examination
E33	Evaluation of ChatGPT's performance on emergency medicine board examination questions	2026	Turkish Journal of Emergency Medicine	10.4103/tjem.tjem_262_25	Examination-performance evaluation; direct EM assessment evidence	Türkiye	ChatGPT accuracy and performance on emergency medicine board examination questions
E34	Evaluation of correctness and reliability of GPT, Bard, and Bing chatbots' responses in basic life support scenarios	2025	Sci Rep	10.1038/s41598-024-82948-w	Comparative scenario-response evaluation; direct BLS education evidence	Iran	Correctness and reliability of chatbot responses in basic life support scenarios
E35	Evaluation of the accuracy and safety of machine translation of patient-specific discharge instructions: a comparative analysis	2026	BMJ quality & safety	10.1136/bmjqs-2024-018384	Comparative translation accuracy/safety evaluation; indirect discharge-communication education evidence	United States / discharge-instruction machine translation	Translation accuracy, safety, and clinical suitability of patient-specific discharge instructions

E36	How well does GPT-4 perform on an emergency medicine board exam? A comparative assessment	2025	Cjem	10.1007/s43678-025-00951-0	Comparative examination-performance assessment; direct EM board-examination evidence	Canada	GPT-4 performance on emergency medicine board examination questions
E37	Large Language Model (LLM)-Powered Chatbots Fail to Generate Guideline-Consistent Content on Resuscitation and May Provide Potentially Harmful Advice	2023	Prehospital and disaster medicine	10.1017/s1049023x23006568	Guideline-concordance and safety evaluation; direct resuscitation education evidence	Not country-specific / resuscitation-guideline chatbot benchmark	Guideline consistency and potential harmful advice in resuscitation-related content
E38	Large Language Model Assistant for Emergency Department Discharge Documentation	2025	JAMA Netw Open	10.1001/jamanetworkopen.2025.38427	LLM documentation assistant evaluation; indirect ED documentation education evidence	South Korea / ED discharge documentation	Discharge documentation quality, completeness, workflow feasibility, and education/coaching relevance
E39	Mapping artificial intelligence models in emergency medicine: A scoping review on artificial intelligence performance in emergency care and education	2025	Turkish Journal of Emergency Medicine	10.4103/tjem.tjem_45_25	Scoping review / evidence map; EM care and education evidence synthesis	Not country-specific / scoping review	Mapping of AI model performance across emergency care and education domains
E40	Perceptions of large language models in medical education and clinical practice among pediatric emergency physicians in Saudi Arabia: a multiregional cross-sectional study	2025	Frontiers in Public Health	10.3389/fpubh.2025.1634638	Multiregional cross-sectional survey; perceptions/implementation evidence	Saudi Arabia	Perceptions, acceptance, concerns, and educational/clinical use of LLMs among pediatric emergency physicians
E41	Performance of ChatGPT in emergency medicine residency exams in Qatar: A comparative analysis with resident physicians	2024	Qatar Medical Journal	10.5339/qmj.2024.61	Comparative examination-performance study; direct EM residency assessment evidence	Qatar	ChatGPT performance relative to emergency medicine residents on residency examination items
E42	Practical Applications of Generative Artificial Intelligence for Clinical Teaching in the Emergency Department	2026	AEM Education and Training	10.1002/aet2.70146	Educational practice/implementation article; direct ED clinical-teaching evidence	United States / ED clinical teaching guidance*	Practical teaching applications, educational workflows, and safeguards for generative AI in the ED
E43	Proof of Concept: Using ChatGPT to Teach Emergency Physicians How to Break Bad News	2023	Cureus	10.7759/cureus.38755	Proof-of-concept educational study; direct communication-training evidence	United States / breaking-bad-news proof-of-concept*	Feasibility and educational value of using ChatGPT to teach breaking-bad-news communication
E44	Simulation and education Testing ChatGPT ability to answer laypeople questions about cardiac arrest and cardiopulmonary resuscitation	2024	Resuscitation	10.1016/j.resuscitation.2023.110077	Simulation/education question-answer evaluation; public CPR education evidence	Italy / United Kingdom / public CPR Q&A context*	Accuracy, appropriateness, and educational quality of answers to layperson cardiac arrest/CPR questions
E45	Suitability of GPT-4o as an evaluator of cardiopulmonary resuscitation skills examinations	2024	Resuscitation	10.1016/j.resuscitation.2024.110404	Evaluator-validity study; direct CPR skills-assessment evidence	China / CPR skills examination evaluation*	Suitability, agreement, and validity of GPT-4o as an evaluator of CPR skills examinations
E46	The Role of Large Language Models in Transforming Emergency Medicine: Scoping Review	2024	JMIR Med Inform	10.2196/53787	Scoping review; direct/indirect EM LLM evidence synthesis	Not country-specific / scoping review	Scope, roles, benefits, limitations, and educational implications of LLMs in emergency medicine
E47	Using participatory action research to develop an artificial intelligence-augmented, peer-driven, case-based, and simulation-enhanced curriculum for emergency medicine residents	2026	Canadian Journal of Emergency Medicine / CJEM	10.1007/s43678-026-01118-1	Participatory action research / curriculum development; direct EM residency education evidence	Canada	Development, feasibility, and acceptability of an AI-augmented, peer-driven, case-based, simulation-enhanced curriculum
E48	Will code one day run a code? Performance of language models on ACEM primary examinations and implications	2023	Emergency Medicine Australasia	10.1111/1742-6723.14280	Language-model examination-performance benchmark; direct ACEM examination evidence	Australia / Australasian College for Emergency Medicine examination	Performance of language models on ACEM primary examination items and educational implications

C1	Application of virtual reality technology in adult CPR training:a scoping review	2024	Chinese Journal of Critical Care Medicine	10.3969/j.issn.1002-1949.2024.01.012	Scoping review; direct adult CPR virtual-reality training evidence	China	Evidence map of virtual reality technology applications in adult CPR training
C2	The opportunities and challenges brought by large-scale natural language models represented by DeepSeek to the standardized training of resident physicians in the emergency medicine department	2025	Chinese Journal of Emergency Medicine	10.3760/cma.j.issn.1671-0282.2025.05.002	Commentary/review; direct EM standardized residency training evidence	China	Opportunities, challenges, and training implications of DeepSeek and natural-language LLMs
C3	Research progress of High-Fidelity Simulation Teaching in clinical emergency nursing	2025	Capital Food Medicine	10.3969/j.issn.1005-8257.2025.15.006	Narrative review / research-progress summary; direct emergency nursing simulation education evidence	China	Progress, applications, and educational implications of high-fidelity simulation teaching in emergency nursing
C4	Application of a virtual simulation system for chemical defense treatment in chemical defense training	2026	China Journal of Emergency Resuscitation and Disaster Medicine	10.3969/j.issn.1673-6966.2026.03.026	Application/evaluation of virtual simulation system; direct disaster/chemical-defense training evidence	China	Training application and potential educational value of virtual simulation for chemical defense treatment
C5	Research progress overview and prospects of post-disaster psychological first aid training based on virtual simulation technology	2024	West China Medical Journal	10.7507/1002-0179.202404213	Narrative review / progress overview; direct psychological first-aid training evidence	China	Research progress and prospects of virtual simulation technology for post-disaster psychological first aid training

F. Supplementary Table S6. Study characteristics and evidence focus

ID	Original title / Excel title	LLM / AI system	Participants / population	Educational context
E1	A comparative analysis of the performance of large language models in the basic life support exam: Comprehensive evaluation of ChatGPT-4o, Gemini 2.0, Claude 3.5, and DeepSeek R1	ChatGPT-4o; ChatGPT; Gemini; Claude; DeepSeek	Resuscitation/BLS/CPR training or guideline education	Resuscitation/BLS/CPR training or guideline education
E2	Advanced Prompt Engineering in Emergency Medicine and Anesthesia: Enhancing Simulation-Based e-Learning	AI / XR / virtual simulation	Emergency medicine/ED learners or clinicians	Emergency medicine/ED learners or clinicians
E3	AI performance in emergency medicine fellowship examination: comparative analysis of ChatGPT-4o, Gemini 2.0, Claude 3.5, and DeepSeek R1 models	ChatGPT-4o; ChatGPT; Gemini; Claude; DeepSeek; AI / XR / virtual simulation	Emergency medicine/ED learners or clinicians	Emergency medicine/ED learners or clinicians
E4	AI versus human-generated multiple-choice questions for medical education: a cohort study in a high-stakes examination	AI / XR / virtual simulation	Emergency medicine/ED learners or clinicians	Emergency medicine/ED learners or clinicians
E5	An AI-Driven Trainee Performance Evaluation in XR-Based CPR Training System for Enhancing Personalized Proficiency	AI / XR / virtual simulation	Resuscitation/BLS/CPR training or guideline education; Emergency medicine/ED learners or clinicians	Resuscitation/BLS/CPR training or guideline education; Emergency medicine/ED learners or clinicians
E6	Artificial intelligence and informatics in neonatal resuscitation	AI / XR / virtual simulation	Resuscitation/BLS/CPR training or guideline education; Neonatal resuscitation education	Resuscitation/BLS/CPR training or guideline education; Neonatal resuscitation education

E7	Artificial intelligence based resuscitation simulation: a pilot study of a novel approach to team leadership training	AI / XR / virtual simulation	Resuscitation/BLS/CPR training or guideline education; Emergency medicine/ED learners or clinicians; Team leadership training	Resuscitation/BLS/CPR training or guideline education; Emergency medicine/ED learners or clinicians; Team leadership training
E8	Assessment and Integration of Large Language Models for Automated Electronic Health Record Documentation in Emergency Medical Services	Large language model(s)	Emergency medicine/ED learners or clinicians; Documentation, handoff, or discharge communication education	Emergency medicine/ED learners or clinicians; Documentation, handoff, or discharge communication education
E9	AutoCLC: Towards Automated Assessment and Feedback on Closed-Loop Communication in Team-based Healthcare Simulation Training	AI / XR / virtual simulation	Emergency medicine/ED learners or clinicians	Emergency medicine/ED learners or clinicians
E10	Automatic Generation of Learning Material for Residential Students in ED: Prompt Engineering and Evaluation	AI/LLM tool not specified in title/source	Emergency medicine/ED learners or clinicians; Residents/trainees	Emergency medicine/ED learners or clinicians; Residents/trainees
E11	Beyond the chain of survival: a scoping review of artificial intelligence applications in cardiac arrest	AI / XR / virtual simulation	Resuscitation/BLS/CPR training or guideline education	Resuscitation/BLS/CPR training or guideline education
E12	Can DALL-E 3 Reliably Generate 12-Lead ECGs and Teaching Illustrations?	DALL-E 3	Emergency or acute-care education context; details require full-text verification	Emergency or acute-care education context; details require full-text verification
E13	ChatG-PD? Comparing large language model artificial intelligence and faculty rankings of the competitiveness of standardized letters of evaluation	ChatGPT; AI / XR / virtual simulation	Emergency medicine/ED learners or clinicians	Emergency medicine/ED learners or clinicians
E14	ChatGPT for use in technology-enhanced learning in anesthesiology and emergency medicine and potential clinical application of AI language models. Between hype and reality around artificial intelligence in medical use	ChatGPT; AI / XR / virtual simulation	Emergency medicine/ED learners or clinicians	Emergency medicine/ED learners or clinicians
E15	ChatGPT's Performance in Cardiac Arrest and Bradycardia Simulations Using the American Heart Association's Advanced Cardiovascular Life Support Guidelines: Exploratory Study	ChatGPT; AI / XR / virtual simulation	Resuscitation/BLS/CPR training or guideline education; Emergency medicine/ED learners or clinicians	Resuscitation/BLS/CPR training or guideline education; Emergency medicine/ED learners or clinicians
E16	Comparative Analysis of Artificial Intelligence-Generated and Human-Written Personal Statements in Emergency Medicine Applications	AI / XR / virtual simulation	Emergency medicine/ED learners or clinicians	Emergency medicine/ED learners or clinicians
E17	Comparative performance of ChatGPT, Gemini, and final-year emergency medicine clerkship students in answering multiple-choice questions: implications for the use of AI in medical education	ChatGPT; Gemini; AI / XR / virtual simulation	Emergency medicine/ED learners or clinicians; Clerkship students	Emergency medicine/ED learners or clinicians; Clerkship students
E18	Comparison of AI-generated and clinician-designed multiple-choice questions in emergency medicine exam: a psychometric analysis	AI / XR / virtual simulation	Emergency medicine/ED learners or clinicians	Emergency medicine/ED learners or clinicians
E19	Comparison of performance of artificial intelligence tools in answering emergency medicine question pool: ChatGPT 4.0, Google Gemini and Microsoft Copilot	ChatGPT; Gemini; Copilot; AI / XR / virtual simulation	Emergency medicine/ED learners or clinicians	Emergency medicine/ED learners or clinicians
E20	Completeness and readability of GPT-4-generated multilingual discharge instructions in the pediatric emergency department	GPT-4	Emergency medicine/ED learners or clinicians; Pediatric emergency setting; Documentation, handoff, or discharge communication education	Emergency medicine/ED learners or clinicians; Pediatric emergency setting; Documentation, handoff, or discharge communication education

E21	Conversational AI in tactical combat casualty care: Baseline GPT-4o improves medic decision-making	ChatGPT-4o; AI / XR / virtual simulation	Emergency medicine/ED learners or clinicians; EMS/prehospital or tactical care personnel	Emergency medicine/ED learners or clinicians; EMS/prehospital or tactical care personnel
E22	Custom GPTs Enhancing Performance and Evidence Compared with GPT-3.5, GPT-4, and GPT-4o? A Study on the Emergency Medicine Specialist Examination	ChatGPT-4o; GPT-4; GPT-3.5; Custom GPTs	Emergency medicine/ED learners or clinicians	Emergency medicine/ED learners or clinicians
E23	Developing and Evaluating Large Language Model-Generated Emergency Medicine Handoff Notes	Large language model(s)	Emergency medicine/ED learners or clinicians; Documentation, handoff, or discharge communication education	Emergency medicine/ED learners or clinicians; Documentation, handoff, or discharge communication education
E24	Differential performance of large language models in advanced cardiac life support assessment: A comprehensive multi-dimensional analysis of accuracy, consistency, and visual recognition capabilities	Large language model(s)	Emergency medicine/ED learners or clinicians	Emergency medicine/ED learners or clinicians
E25	Effectiveness of ChatGPT for Clinical Scenario Generation: A Qualitative Study	ChatGPT	Emergency or acute-care education context; details require full-text verification	Emergency or acute-care education context; details require full-text verification
E26	Embracing Large Language Models for Adult Life Support Learning	Large language model(s)	Resuscitation/BLS/CPR training or guideline education	Resuscitation/BLS/CPR training or guideline education
E27	Empowering Women in Academic Emergency Medicine: A Time-Efficient Workshop Incorporating Large Language Models to Enhance Leadership Skills	Large language model(s)	Emergency medicine/ED learners or clinicians; Team leadership training; Faculty/academic leadership development	Emergency medicine/ED learners or clinicians; Team leadership training; Faculty/academic leadership development
E28	Evaluating Artificial Intelligence Competency in Education: Performance evaluation of ChatGPT in the neonatal resuscitation program exam	ChatGPT; AI / XR / virtual simulation	Resuscitation/BLS/CPR training or guideline education; Neonatal resuscitation education; Emergency medicine/ED learners or clinicians	Resuscitation/BLS/CPR training or guideline education; Neonatal resuscitation education; Emergency medicine/ED learners or clinicians
E29	Evaluating the accuracy and reliability of AI chatbots in disseminating the content of current resuscitation guidelines: a comparative analysis between the ERC 2021 guidelines and both ChatGPTs 3.5 and 4	ChatGPT; AI / XR / virtual simulation	Resuscitation/BLS/CPR training or guideline education	Resuscitation/BLS/CPR training or guideline education
E30	Evaluation of a Large Language Model on the American Academy of Pediatrics' PREP Emergency Medicine Question Bank	Large language model(s)	Emergency medicine/ED learners or clinicians; Pediatric emergency setting	Emergency medicine/ED learners or clinicians; Pediatric emergency setting
E31	Evaluation of ChatGPT Performance on Emergency Medicine Board Examination Questions: Observational Study	ChatGPT	Emergency medicine/ED learners or clinicians	Emergency medicine/ED learners or clinicians
E32	Evaluation of ChatGPTs Performance in Türkiye's First Emergency Medicine Sub-Specialization Exam	ChatGPT	Emergency medicine/ED learners or clinicians; Türkiye setting	Emergency medicine/ED learners or clinicians; Türkiye setting
E33	Evaluation of ChatGPT's performance on emergency medicine board examination questions	ChatGPT	Emergency medicine/ED learners or clinicians	Emergency medicine/ED learners or clinicians
E34	Evaluation of correctness and reliability of GPT, Bard, and Bing chatbots' responses in basic life support scenarios	Bard; Bing	Resuscitation/BLS/CPR training or guideline education	Resuscitation/BLS/CPR training or guideline education

E35	Evaluation of the accuracy and safety of machine translation of patient-specific discharge instructions: a comparative analysis	Machine translation	Documentation, handoff, or discharge communication education	Documentation, handoff, or discharge communication education
E36	How well does GPT-4 perform on an emergency medicine board exam? A comparative assessment	GPT-4	Emergency medicine/ED learners or clinicians	Emergency medicine/ED learners or clinicians
E37	Large Language Model (LLM)-Powered Chatbots Fail to Generate Guideline-Consistent Content on Resuscitation and May Provide Potentially Harmful Advice	Large language model(s); AI / XR / virtual simulation	Resuscitation/BLS/CPR training or guideline education; Emergency medicine/ED learners or clinicians	Resuscitation/BLS/CPR training or guideline education; Emergency medicine/ED learners or clinicians
E38	Large Language Model Assistant for Emergency Department Discharge Documentation	Large language model(s)	Emergency medicine/ED learners or clinicians; Documentation, handoff, or discharge communication education	Emergency medicine/ED learners or clinicians; Documentation, handoff, or discharge communication education
E39	Mapping artificial intelligence models in emergency medicine: A scoping review on artificial intelligence performance in emergency care and education	AI / XR / virtual simulation	Emergency medicine/ED learners or clinicians	Emergency medicine/ED learners or clinicians
E40	Perceptions of large language models in medical education and clinical practice among pediatric emergency physicians in Saudi Arabia: a multiregional cross-sectional study	Large language model(s)	Emergency medicine/ED learners or clinicians; Pediatric emergency setting; Saudi Arabia setting	Emergency medicine/ED learners or clinicians; Pediatric emergency setting; Saudi Arabia setting
E41	Performance of ChatGPT in emergency medicine residency exams in Qatar: A comparative analysis with resident physicians	ChatGPT	Emergency medicine/ED learners or clinicians; Residents/trainees; Qatar setting	Emergency medicine/ED learners or clinicians; Residents/trainees; Qatar setting
E42	Practical Applications of Generative Artificial Intelligence for Clinical Teaching in the Emergency Department	AI / XR / virtual simulation	Emergency medicine/ED learners or clinicians	Emergency medicine/ED learners or clinicians
E43	Proof of Concept: Using ChatGPT to Teach Emergency Physicians How to Break Bad News	ChatGPT	Emergency medicine/ED learners or clinicians	Emergency medicine/ED learners or clinicians
E44	Simulation and education Testing ChatGPT ability to answer laypeople questions about cardiac arrest and cardiopulmonary resuscitation	ChatGPT; AI / XR / virtual simulation	Resuscitation/BLS/CPR training or guideline education; Emergency medicine/ED learners or clinicians; Public/layperson emergency education	Resuscitation/BLS/CPR training or guideline education; Emergency medicine/ED learners or clinicians; Public/layperson emergency education
E45	Suitability of GPT-4o as an evaluator of cardiopulmonary resuscitation skills examinations	ChatGPT-4o	Resuscitation/BLS/CPR training or guideline education	Resuscitation/BLS/CPR training or guideline education
E46	The Role of Large Language Models in Transforming Emergency Medicine: Scoping Review	Large language model(s)	Emergency medicine/ED learners or clinicians	Emergency medicine/ED learners or clinicians
E47	Using participatory action research to develop an artificial intelligence-augmented, peer-driven, case-based, and simulation-enhanced curriculum for emergency medicine residents	AI / XR / virtual simulation	Emergency medicine/ED learners or clinicians; Residents/trainees	Emergency medicine/ED learners or clinicians; Residents/trainees
E48	Will code one day run a code? Performance of language models on ACEM primary examinations and implications	AI/LLM tool not specified in title/source	Australasian emergency medicine examination setting	Australasian emergency medicine examination setting

C1	Application of virtual reality technology in adult CPR training:a scoping review	Virtual reality / AI-adjacent simulation technology	Adult CPR training; emergency/resuscitation education	Adult CPR training; emergency/resuscitation education
C2	The opportunities and challenges brought by large-scale natural language models represented by DeepSeek to the standardized training of resident physicians in the emergency medicine department	DeepSeek and natural-language LLMs	Emergency medicine standardized residency training	Emergency medicine standardized residency training
C3	Research progress of High-Fidelity Simulation Teaching in clinical emergency nursing	High-fidelity simulation / AI-adjacent technology	Emergency nursing simulation training	Emergency nursing simulation training
C4	Application of a virtual simulation system for chemical defense treatment in chemical defense training	Virtual simulation system	Chemical defense/disaster emergency training	Chemical defense/disaster emergency training
C5	Research progress overview and prospects of post-disaster psychological first aid training based on virtual simulation technology	Virtual simulation technology	Post-disaster psychological first aid training	Post-disaster psychological first aid training
